# Comparison of CHROMagar, polymerase chain reaction-restriction fragment length polymorphism, and polymerase chain reaction-fragment size for the identification of Candida species 

**DOI:** 10.29252/cmm.3.3.10

**Published:** 2017-09

**Authors:** Zahra Jafari, Marjan Motamedi, Nilufar Jalalizand, Gholam. R Shokoohi, Arezu Charsizadeh, Hossein Mirhendi

**Affiliations:** 1 Department of Medical Parasitology and Mycology, School of Public Health, Tehran University of Medical Sciences, Tehran, Iran; 2 Department of Medical Parasitology and Mycology, School of Medicine, Shiraz University of Medical Sciences, Shiraz, Iran; 3 Department of Medical Parasitology and Mycology, School of Public Health, National Institute of Health Research, Tehran University of Medical Sciences, Tehran, Iran; 4 Department of Medical Parasitology and Mycology, School of Medicine, Jahrom University of Medical Sciences, Jahrom, Iran; 5 Department of Medical Parasitology and Mycology, School of Medicine, Isfahan University of Medical Sciences, Isfahan, Iran

**Keywords:** *Candida* species, Identification, PCR-fragment size polymorphism, PCR-restriction fragment length polymorphism

## Abstract

**Background and Purpose:**

The epidemiological alteration in the distribution of *Candida *species, as well as the significantly increasing trend of either intrinsic or acquired resistance of some of these fungi highlights the need for a reliable method for the identification of the species. Polymerase chain reaction (PCR) is one of the methods facilitating the quick and precise identification of *Candida *species. The aim of this study was to compare the efficiency of CHROMagar, PCR-restriction fragment length polymorphism (PCR-RFLP), and PCR-fragment size polymorphism (PCR-FSP) assays in the identification of *Candida* species to determine the benefits and limitations of these methods.

**Materials and Methods:**

This study was conducted on 107 *Candida* strains, including 20 standard strains and 87 clinical isolates. The identification of the isolates was accomplished by using CHROMagar as a conventional method. The PCR-RFLP assay was performed on the entire internal transcribed spacer (ITS) region of ribosomal DNA (rDNA), and the consequent enzymatic digestion was compared with PCR-FSP results in which ITS1 and ITS2 regions were separately PCR amplified. In both molecular assays, yeast identification was carried out through the specific electrophoretic profiles of the PCR products.

**Results:**

According to the results, the utilization of CHROMagar resulted in the identification of 29 (33.3%) *Candida* isolates, while the PCR-RFLP and PCR-FSP facilitated the identification of 83 (95.4%) and 80 (91.9%) clinical isolates, respectively. The obtained concordances between CHROMagar and PCR-RFLP, between CHROMagar and PCR-FSP, as well as between PCR-RFLP and PCR-FSP were 0.23, 0.20, and 0.77, respectively.

**Conclusion:**

The recognition of the benefits and limitations of PCR methods allows for the selection of the most efficient technique for a fast and correct differentiation. The PCR-RFLP and PCR-FSP assays had satisfactory concordance. The PCR-FSP provides a rapid, technically simple, and cost-effective method for the identification of *Candida* species. Nevertheless, to accurately differentiate among the taxonomically related species, PCR-RFLP should be implemented.

## Introduction

*Candida *species usually reside as commensals at mucosal membranes in healthy individuals and can be detected in approximately 50% of the population in a non-virulent form [1]. However, these species can become pathogenic in case the host’s normal flora is disrupted or the immunity is impaired. Among the human pathogenic fungi, genus *Candida *has a dominant role in afflicting the hospitalized patients with systemic life-threatening infections [2]. *Candida *species has been the fourth most common microorganisms causing nosocomial blood stream infection in hospitalized patients [3]. This genus includes at least 30 species of medical importance that are involved in human candidiasis [4].

During the past several decades, the incidence of infections caused by genus *Candida* has substantially increased due to the expansion of immunosuppressive situations. Moreover, those species once thought tobe non-pathogenic are currently considered as opportunistic pathogens [5, 6]. The current changes in the epidemiology of candidiasis highlight a shift in the prevalence of *Candida* species so that a reduced proportion of *C. albicans* and an increase in non-*albicans* *Candida *species Can be seen [7]. Based on a recent review, while in the 1980s, *C. albicans* accounted for more than 80% of all *Candida* isolates recovered from nosocomial yeast infection [8], currently, *C. albicans*  constitutes less than 50% of all *Candida* blood isolates [9]. 

Considering the variation of *Candida* species in susceptibility to antifungal agents, the rapid and accurate identification of the species may assist in finding an appropriate therapy for candidiasis [10]. On the other hand, there is a need for recognizing the main source of the infections and determine whether itis endogenous or acquired exogenously from other patients or even health care workers [11, 12]. Therefore, the precise identification of the strains at species and sub-species levels is highly demanded to perform epidemiological investigations and control the outbreaks. 

There are many assays targeted toward the identification of *Candida *species, which could be divided into two phenotypic and genotypic groups. Phenotypic assays, such as yeast colony morphologies on malt extract agar or chromogenic culture media, sugar absorption and fermentation tests, and commercial kits (e.g., API), can be time-consuming. Furthermore, the reliance of these techniques on the variable expression of phenotypic characteristics can lead to inconsistent results. 

In contrast, genotypic assays that are mostly DNA-based approaches are more accurate and less vulnerable to variations due to growth condition and phenotypic switching. Among the various molecular techniques, the best known methods include specific primers in polymerase chain reaction (PCR) and multiplex PCR [13], specific probes for each species [14], PCR-restriction fragments length polymorphism (PCR-RFLP) [15], sequencing of specific regions of genome [16], real-time PCR [17], and PCR-fragment size polymorphism (PCR-FSP) [18]. 

There are several studies confirming PCR-RFLP [19-21] and PCR-FSP [18, 22] assays as simple, rapid, inexpensive, and highly valuable tools, which can be used to differentiate the *Candida* species. Therefore, in this present study, we aimed to compare the efficiency of these PCR assays and CHROMagar assay as a conventional method in terms of performance, accuracy, speed, and cost.

## Materials and Methods

This study was conducted on a total of 107 *Candida* strains, including 20 standard strains and 87 clinical isolates. The standard strains were supplied by Teikyo University Institute of Medical Mycology (TIMM), Tokyo, Japan. These strains included *C. albicans* (TIMM 1768), *C. krusei* (TIMM 3404), *C. kefyr* (TIMM 0300), *C. tropicalis* (TIMM 0313), *C. guilliermondii* (TIMM 3400), *C. lusitaniae* (TIMM 1439), and *C. rugosa* (TIMM 3411). The clinical strains used in the study were part of a large collection already isolated from various clinical specimens obtained from different hospitals in Tehran, Iran [23]. 

The isolates were subcultured on Sabouraud Dextros agar (Difco, Detroit, MI, USA) containing Chloramphenicol (Merck, Germany) and incubated for 2 days at 30°C to obtain *Candida* colony. For the identification of *Candida* strains by means of CHROMagar, each isolate was subcultured on a CHROMagar *Candida* (CHROMagar, Paris, France) plate and incubated at 35°C for 48 h. *Candida* species were identified based on the colony color according to the manufacturer’s instructions. For DNA extraction, the genomic DNA of each yeast was extracted by boiling assay. Briefly, a single colony was re-suspended in 50 μL sterile water, heated for 10 min at 95°C, and centrifuged for 3 min at 2000 g. The obtained supernatant was preserved at -20°C until use.

In order to identify the *Candida* species by PCR-RFLP, the primers ITS1 (5’-TCC GTA GGT GAA CCT GCG G-3’) and ITS4 (5’-TCC TCC GCT TAT TGA TAT GC-3’) were used to amplify contiguous ITS1-5.8SrDNA-ITS2 region of the rDNA genes in the PCR reaction, followed by digestion by the restriction enzyme *MSP*1 as described before [23]. Furthermore, for species identification by PCR-FSP, the ITS1 and ITS2 regions were simultaneously PCR-amplified in separate reaction tubes with the ITS1-ITS2 (5’- GCTGCGTTCTTCATCGATGC-3’) and ITS3-ITS4 (5’ GCATCGATGAAGAACGCAGC- 3’) primer sets, respectively, as already described [18]. 

Subsequently, 3 μL of the PCR-RFLP products and each PCR-FSP amplicons were separated on 2% agarose gel electrophoresis in TBE buffer (Tris 90mM, Boric acid 90mM, EDTA 2mM) for about 2.5 h at 80 V. Species identification was based on the unique pattern of each species. The created bands were detected by staining with 0.5 μg/mL of ethidium bromide, and then photographed. *Candida* species were identified by the two PCR methods according to the expected band size obtained from in silico sequencing analysis (Table 1).

**Table 1:** Size of ITS region before and after endonuclease digestion with *MspI* as well as ITS1 and ITS2 fragments for common and rare pathogenic *Candida* species

***Candida *** **species**	**Length of ITS**	**Fragments’ length after enzymatic digestion (** ***MSP*** **1)**	**Length of ITS1 and ITS2 fragments**	
			**ITS2**	**ITS1**
*Candida albicans*			214	**ITS2**
*Candida parapsilosis*	537	239, 298	225	340
*Candida glabrata*	530	530	475	309
*Candida rugosa*	881	320, 561	141	413
*Candida guilliermondii*	399	121, 278	243	270
*Candida kefyr*	607	82, 155, 370	305	374
*Candida lusitaniae*	720	720	145	427
*Candida famata*	382	118, 264	-	251
*Candida tropicalis*	639	639	214	-
*Candida krusei*	*526*	186, 340	181	327
	510	250, 260	214	344

**Table 2:** Comparison of agreement between the assays

**Test**	**Group 1**	**Group 2**
CHROMagar and PCR-RFLP	κ=0.89	κ=0.23
CHROMagar and PCR-FSP	κ=0.87	κ=0.20
PCR-RFLP and PCR-FSP	κ=0.97	κ=0.77

Group 1: species identified by the three assays, Group 2: species identified by the two molecular assays.

***Statistical analysis***

In order to measure the agreement between the assays, the samples were classified into two groups (Table 2). One group was comprised of the species that could be identified by both genotypic and phenotypic approaches, and the other group contained the species identified only by the molecular assays. The agreement was analyzed using the Cohen’s kappa (k) coefficient. Data analysis was performed in SPSS, version 16.0.

## Results

The standard strains, including a vast variety of yeast species, were subjected to species identification using CHROMagar, PCR-RFLP, and PCR-FSP assays. Although there was no disagreement amongthe assays regarding the identification capability, CHROMagar was unable to identify many species. The frequency rates of identification of the 87 clinical samples by the three investigated assays are presented in Table 3. When it was not possible to determine the species of a strain, the result was classified as *Candida* species.

**Figure 1:**
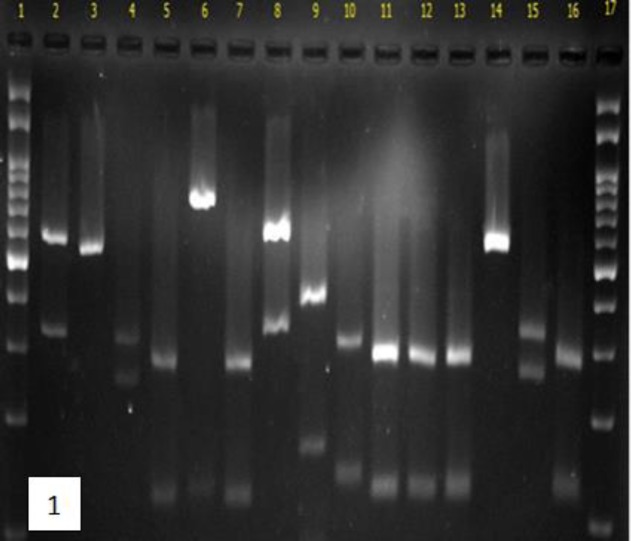
Restriction digestion by the *Msp1* enzyme of PCR products of some *Candida* strains in PCR- RFLP; lanes 1 and 17 are 100 bp DNA markers, lanes 2-16 are *C. glabrata*, *C. parapsilosis*, *C. albicans*, *C. lusitaniae*, *C. famata*, *C. lusitaniae*, *C. glabrata*, *C. guilliermondii*, *C. rugosa*, *C. lusitaniae*, *C. lusitaniae*, *C. lusitaniae*, *C. famata*, *C. albicans*, and *C. rugose*, respectively.

**Figure 2:**
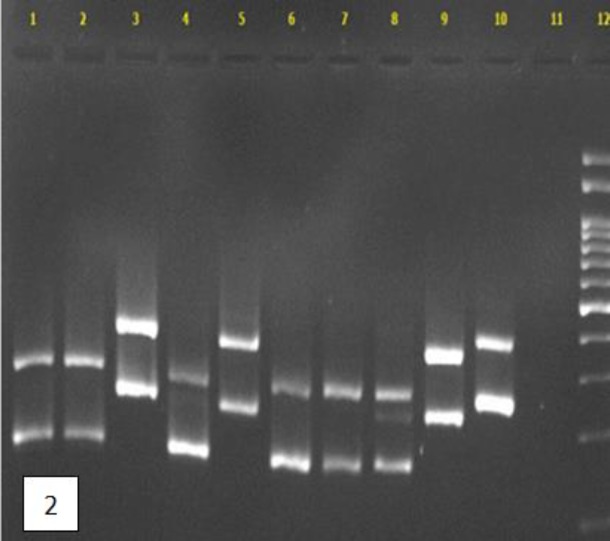
Agarose gel electrophoresis of mixed ITS1 and ITS2 PCR products of some *Candida* strains in PCR-FSP; lanes 1-10 are *C. rugosa*, *C. rugosa*, *C. guilliermondii*, *C. lusitaniae*, *C. albicans*, C*. rugosa*, *C. rugosa*, *C. rugosa*, *C. albicans*, and *C. guilliermondii* respectively, lane 11 is negative control, and lane 12 is 100 bp DNA size marker.

According to the results, CHROMagar could identify 29 (33.3%) *Candida* isolates, while PCR-RFLP and PCR-FSP identified 83 (95.4%) and 80 (91.9%) isolates, respectively. The PCR-RFLP was able to identify the most diverse species (10 species), followed by PCR-FSP and CHROMagar (9 and 3 species, respectively). Figure 1 demonstrates an example of the variation of *Candida* species identified by PCR-RFLP after digestion by the *MSP1* enzyme. The agarose gel electrophoresis of mixed ITS1 and ITS2 PCR amplicons in PCR-FSP is illustrated in Figure 2.

The comparison of the agreement between the assays is described in Table 2. Considering the species types in Group 1, namely *C. albicans*, *C. tropicali*s, *C. krusei,* and *Candida* species, the agreement values between CHROMagar and PCR-RFLP, CHROMagar and PCR-FSP, and PCR-RFLP and PCR-FSP were 0.89, 0.87, and 0.97, respectively. Therefore, all assays had a satisfactory concordance. In Group 2, which included all species identified by the two molecular assays, there were slight (κ=0.23), considerable (κ=0.20), and substantial (κ=0.77) concordances between CHROMagar and PCR-RFLP, between CHROMagar and PCR-FSP, as well as between PCR-RFLP and PCR-FSP, respectively.

The minimum time needed for the identification of 10 samples of yeast species in our study included approximately 2 days for CHROMagar, 8 h for PCR-RFLP, and 6 h for PCR-FSP.

**Table 3:** Identification of clinical *Candida* isolates by CHROMagar, PCR-RFLP, and PCR-FSP assays

**Species**	**CHROMagar **	**PCR-RFLP**	**PCR-FSP**
*Candida albicans*	24 (27.6%)	22 (25.3%)	22 (25.3%)
*Candida parapsilosis*	-	15 (17.2%)	15 (17.2%)
*Candida glabrata*	-	15 (17.2%)	16 (18.4%)
*Candida rugosa*	-	14 (16.1%)	8 (9.2%)
*Candida guilliermondii*	-	5 (5.7%)	5 (5.7%)
*Candida kefyr*	-	4 (4.6%)	2 (2.3%)
*Candida lusitaniae*	-	3 (3.4%)	1 (1.1%)
*Candida famata*	-	1 (1.1%)	-
*Candida tropicalis*	4 (4.6%)	2 (2.3%)	2 (2.3%)
*Candida krusei*	1 (1.1%)	1 (1.1%)	2 (2.3%)
* Candida rugosa or lusitaniae*	-	1 (1.1%)	7 (8.0%)
*Candida* spp.	58 (66.7%)	4 (4.6%)	7 (8.0%)
Total	87 (100%)	87 (100%)	87 (100%)

## Discussion

The rapid and accurate identification of the clinical isolates of *Candida* species can affect the mortality rate, cost of treatment, and hospitalization duration for the invasive infections. In this study, the chromogenic medium CHROMagar*, *PCR-RFLP, and PCR-FSP were compared regarding their performance, accuracy, speed, and cost in identifying the *Candida* species. 

We found that although CHROMagar is a straightforward assay, it was unable to recognize more than three species. Accordingly, this method could only identify 33.3% of all the isolates at the species level, while 4.6% and 8.0% of the samples tested by PCR-RFLP and PCR-FSP, respectively, were not identified. Accordingly, there are several similar studies reporting an inconsistency between the results of molecular and phenotypic assays [24, 25].

The chromogenic medium CHROMagar has been provided for both isolation and identification of *Candida* species*,* based on the pigmentation of the colonies with different colors, which is due to different enzyme activities in *Candida *species. This technique has the advantage of being inexpensive and less difficult in comparison with other conventional assays, such as API systems and Vitek 2 ID system. However, this method is time-consuming in comparison to molecular assays, such as PCR-RFLP and PCR-FSP. This medium is able to detect the presence of mixed cultures by giving different colored colonies on a plate at the same time. However, as CHROMagar is designed only for the differentiation of three species )i.e., *C. albicans* [green], *C. tropicali*s [metallic blue with a pink halo], and *C. krusei* [pink with velvety appearance]), it fails to clearly identify other species [26]. Such misclassification of species was also reported in the studies carried out by Estrada et al. and Souza et al. [27, 28]. 

The ability of molecular biology assays to detect fungal pathogens is more reliable than that of the traditional phenotyping assays. The analysis of concordance between PCR-RFLP and PCR-FSP in this study revealed almost a substantial concordance between the two assays (kappa=0.77). In PCR-RFLP assay, considering the size of the fragments obtained from the restriction digestion of the PCR products by an enzyme, the common and also some uncommon or rare pathogenic *Candida* were differentiated. 

The PCR-FSP is also a DNA-based assay for the identification of uncommon pathogenic *Candida* species as well as the common ones by differing in size across one or both ITS1 and ITS2 regions. Nonetheless, the ability of this method to differentiate between taxonomically related speciesis under dispute because it cannot provide sufficient discriminatory power for these species. As mentioned before [18], this approach could not easily distinguish between *C. albicans* and *C. tropicalis*, which are the common causes of candidiasis. 

Moreover, the storage and transfer of the enzyme needed for RFLP could be more expensive. However, PCR-FSP has been demonstrated to be an easy to handle procedure.

On the other hand, the interpretation of the results in PCR-RFLP are fast, easy, and clear, whereas in PCR-FSP assay, it is rather complicated and time-consuming and requires standard conditions for electrophoresis, including having a good marker size and multiple reference controls. In PCR-RFLP, the digestion of the PCR products with restriction enzymes increases the time required to identify *Candida *species, while PCR-FSP does not need post-PCR procedures, such as sequencing, enzymatic digestion, or application of probe. This feature in PCR-FSP would give the clinicians valuable time to decide on the treatment of candidiasis before the antifungal sensitivity reports are available.

While PCR-RFLP requires an enzyme, which may be expensive, PCR-FSP is a cost-effective molecular assay, which requires only basic usual equipment used for PCR and electrophoresis.

Nonetheless, if you need to identify new species by these two PCR methods, adding data to cover its identifications is almost easy in both methods.

## Conclusion

We have described the benefits and limitations of three assays for the identification of *Candida* species. It was concluded that CHROMagar is an excellent assay for the identification of *C. albicans*; nonetheless, additional tests are required for non-*albicans *species other than *C. tropicalis* and *C. krusei*. The PCR assays are more efficient in the identification of other *Candida* species than the CHROMagar; however, the useof each PCR assay has its own advantages and disadvantages. 

Species identification through PCR-FSP is a rapid, technically simple, and cheap procedure as compared to the time-consuming, technically demanding, and expensive PCR-RFLP. The PCR-FSP can be reliably used for the identification of common and also some uncommon species. Nevertheless, the identificationof taxonomically related species requires the implementation of PCR-RFLP. 

## References

[1] Naglik JR, Challacombe SJ, Hube B (2003). Candidaalbicans secreted aspartyl proteinases in virulence and pathogenesis. Microbiol Mol Biol Rev.

[2] Jarvis WR (1995). Epidemiology of nosocomial fungal infections, with emphasis on Candida species. Clin Infect Dis.

[3] Hajjeh RA, Sofair AN, Harrison LH, Lyon GM, Arthington-Skaggs BA, Mirza SA (2004). Incidence of bloodstream infections due to Candida species and in vitro susceptibilities of isolates collected from 1998 to 2000 in a population-based active surveillance program. J Clin Microbiol.

[4] Ostrosky-Zeichner L, Pappas PG (2006). Invasive candidiasis in the intensive care unit. Crit Care Med.

[5] Aikawa NE, Rosa DT, Del Negro GM, Moraes JC, Ribeiro AC, Saad CG (2015). Systemic and localized infection by Candida species in patients with rheumatic diseases receiving anti-TNF therapy. Rev Bras Reumatol.

[6] Chow JK, Golan Y, Ruthazer R, Karchmer AW, Carmeli Y, Lichtenberg D (2008). Factors associated with candidemia caused by non-albicans Candida species versus Candida albicans in the intensive care unit. Clin Infect Dis.

[7] Lewis RE (2009). Overview of the changing epidemiology of candidemia. Curr Med Res Opin.

[8] Beck-Sague C, Jarvis WR (1993). Secular trends in the epidemiology of nosocomial fungal infections in the United States, 1980-1990 National Nosocomial Infections Surveillance System. J Infect Dis.

[9] Rangel-Frausto MS, Wiblin T, Blumberg HM, Saiman L, Patterson J, Rinaldi M (1999). National epidemiology of mycoses survey (NEMIS): variations in rates of bloodstream infections due to Candida species in seven surgical intensive care units and six neonatal intensive care units. Clin Infect Dis.

[10] Pfaller MA, Diekema DJ, Jones RN, Messer SA, Hollis RJ, SENTRY Participants Group (2002). Trends in antifungal susceptibility of Candida spp isolated from pediatric and adult patients with bloodstream infections: SENTRY Antimicrobial Surveillance Program, 1997 to 2000. J Clin Microbiol.

[11] Asmundsdottir LR, Erlendsdottir H, Haraldsson G, Guo H, Xu J, Gottfredsson M (2008). Molecular epidemiology of candidemia: evidence of clusters of smoldering nosocomial infections. Clin Infect Dis.

[12] Neu N, Malik M, Lunding A, Whittier S, Alba L, Kubin C (2009). Epidemiology of candidemia at a children's hospital, 2002 to 2006. Pediatr Infect Dis J.

[13] Liguori G, Di Onofrio V, GallA F, Lucariello A, Albano L, Catania M (2010). Candida albicans identification: comparison among nine phenotypic systems and a multiplex PCR. J Prev Med Hyg.

[14] Elie CM, Lott TJ, Reiss E, Morrison CJ (1998). Rapid identification of Candida species with species-specific DNA probes. J Clin Microbiol.

[15] Mohammadi R, Badiee P, Badali H, Abastabar M, Safa AH, Hadipour M (2015). Use of restriction fragment length polymorphism to identify Candida species, related to onychomycosis. Adv Biomed Res.

[16] Fathi N, Mohammadi R, Tabatabaiefar MA, Ghahri M, Sadrossadati SZ (2016). Sequence-identification of Candida species isolated from candidemia. Adv Biomed Res.

[17] Zhang J, Hung GC, Nagamine K, Li B, Tsai S, Lo SC (2016). Development of Candida-specific Real-Time PCR assays for the detection and identification of eight medically important Candida species. Microbiol Insights.

[18] Khodadadi H, Karimi L, Jalalizand N, Adin H, Mirhendi H (2017). Utilization of size polymorphism in ITS1 and ITS2 regions for identification of pathogenic yeast species. J Med Microbiol.

[19] Mirhendi H, Makimura K, Khoramizadeh M, Yamaguchi H (2006). A one-enzyme PCR-RFLP assay for identification of six medically important Candida species. Nippon Ishinkin Gakkai Zasshi.

[20] Shokohi T, Hashemi Soteh M, Saltanat Pouri Z, Hedayati MT, Mayahi S (2010). Identification of Candida species using PCR-RFLP in cancer patients in Iran. Indian J Med Microbiol.

[21] Mousavi SA, Khalesi E, Bonjar GS, Aghighi S, Sharifi F, Aram F (2007). Rapid molecular diagnosis for-candida species using PCR-RFLP. Biotechnology.

[22] Mirhendi H, Shidfar M, Hashemi J, Moazeni M (2008). Identification of pathogenic Candida species: PCR-fragment size polymorphism (PCR-FSP) assay. Tehran Univ Med J.

[23] Mohammadi R, Mirhendi H, Rezaei-Matehkolaei A, Ghahri M, Shidfar MR, Jalalizand N (2013). Molecular identification and distribution profile of Candida species isolated from Iranian patients. Med Mycol.

[24] Kazemi A, Falahati M, Hajipoor A, Jafari A (2013). Comparison of phenotypic tests and PCR to detect Candida Albicans from vaginal specimens (Tabriz, 2009-2010). Jundishapur J Microbiol.

[25] Ahmad S, Khan Z, Mustafa AS, Khan ZU (2002). Seminested PCR for diagnosis of candidemia: comparison with culture, antigen detection, and biochemical assays for species identification. J Clin Microbiol.

[26] Pfaller MA, Houston A, Coffmann S (1996). Application of CHROMagar Candida for rapid screening of clinical specimens for Candida albicans, Candida tropicalis, Candida krusei, and Candida (Torulopsis) glabrata. J Clin Microbiol.

[27] Souza MN, Ortiz SO, Mello MM, Oliveira Fde M, Severo LC, Goebel CS (2015). Comparison between four usual assays of identification of Candida species. Rev Inst Med Trop Sao Paulo.

[28] Estrada-Barraza D, Dávalos Martinez A, Flores-Padilla L, Mendoza-De Elias R, Sanchez-Vargas LO (2011). Comparison between conventional methods, ChromAgar Candida® and PCR method for the identification of Candida species in clinical isolates. Rev Iberoam Micol.

